# The Efficacy and Safety of Teprotumumab in Thyroid Eye Disease: Evidence from Randomized Controlled Trials

**DOI:** 10.1155/2023/6638089

**Published:** 2023-08-08

**Authors:** Fei Lin, Qiu'e Yao, Bin Yu, Zehui Deng, Jingyue Qiu, Rong He

**Affiliations:** ^1^Department of Pharmacy, The First Affiliated Hospital of Chengdu Medical College, Clinical Medical College, Chengdu Medical College, Chengdu, China; ^2^Department of Pharmacy, Changsha Hospital of Traditional Chinese Medicine (Changsha Eighth Hospital), Changsha, China; ^3^Department of Pharmacy, Mianyang Central Hospital, School of Medicine, University of Electronic Science and Technology of China, Mianyang, China; ^4^Department of Pharmacy, West China Hospital, Sichuan University, Chengdu, China; ^5^Department of Pharmacy, PLA Strategic Support Force Medical Center, Beijing, China; ^6^Department of Respiratory and Critical Care Medicine, The First Affiliated Hospital of Chengdu Medical College, Clinical Medical College, Chengdu Medical College, Chengdu, China

## Abstract

In this study, we conducted a meta-analysis to assess the efficacy and safety of teprotumumab in treating thyroid eye disease. We searched the Cochrane Library, PubMed, and Embase databases from inception to May 25, 2022, and included all randomized controlled trials. Odds ratios (ORs) were calculated using fixed- or random-effect models. A total of three studies involving 341 patients were identified. Overall, the analysis revealed that teprotumumab demonstrated superior integrated proptosis response compared to placebo in both the intention-to-treat (ITT) population (OR = 17.81, 95% CI = [10.32, 30.76], *I*^2^ = 50%) and per-protocol population (OR = 24.53, 95% CI = [12.96, 46.45], *I*^2^ = 14%). Furthermore, patients receiving teprotumumab showed significant improvement in overall response (OR = 8.35, 95% CI = [4.74, 14.71], *I*^2^ = 79%), diplopia response (OR = 5.53, 95% CI = [3.24, 9.44], *I*^2^ = 0%), and achieving a clinical activity score (CAS) of 0 or 1 (OR = 6.26, 95% CI = [3.87, 10.12], *I*^2^ = 0%). Moreover, patients treated with teprotumumab experienced greater improvements in proptosis (MD = −2.49, 95% CI = [−2.54, −2.45], *I*^2^ = 98%) and Graves' ophthalmopathy-specific quality of life (GO-QOL, MD = 11.48, 95% CI = [11.03, 11.93], *I*^2^ = 95%). However, it is important to note that patients receiving teprotumumab had a higher risk of adverse events, including serious adverse events, gastrointestinal adverse reactions, and muscle spasms. In summary, teprotumumab demonstrated greater improvement in proptosis response, proptosis, diplopia response, overall response, GO-QOL, and CAS. Nonetheless, it should be considered that its use is associated with a higher risk of adverse events.

## 1. Introduction

Thyroid eye disease (TED), also known as Graves' ophthalmopathy and thyroid-associated ophthalmopathy (TAO), is an orbital disorder that can affect one or both eyes, and it is a common cause of unilateral and bilateral ophthalmoplegia [1, 2]. TED is a rare autoimmune disease characterized by infiltrative lesions in the posterior and periorbital ocular tissues. It is one of the most frequent extra-thyroidal manifestations of thyroid disease [1, 3]. The primary clinical symptoms include inflammation, protrusion of the eyes, and diplopia. The incidence of TED is relatively high, accounting for approximately 20% of orbital diseases, making it the most prevalent orbital disease among adults. In females, the incidence of TED is 16 per 100,000, while in males, it is 2.9 per 100,000 [4–6]. More than 90% of TED patients also have hyperthyroidism, although some may be hypothyroid or euthyroid. Clinically, TED occurs in 40% of individuals with Graves' disease, and approximately 5% of TED patients develop optic neuropathy, which may require urgent treatment [1, 7].

The European Group on Graves' orbitopathy has recommended intravenous glucocorticoids as the first-line treatment for active, moderate-to-severe TED [3, 8]. Glucocorticoids have been found to effectively reduce orbital inflammation in 50% to 80% of cases. However, some individuals may not respond to this treatment and there are notable side effects associated with long-term or high-dose courses. Additionally, there is an 11% relapse rate after 12 weeks of glucocorticoid treatment [9]. Consequently, the development of new drugs for TED treatment has become necessary. In recent years, several therapeutic targets have been identified. These include inhibiting T cell activation and T cell depletion, B cell depletion, cytokine inhibition, anti-TNF alpha monoclonal antibodies, monoclonal antibodies specific for insulin-like growth factor I receptor (IGF-1R), thyroid-stimulating hormone receptor (TSHR) inhibitors, CD40 monoclonal antibodies, and PI3K intracellular pathway inhibitors. The objective of these treatments is to reverse or inhibit the underlying pathophysiology of the disease [8, 9]. Thyroid autoantibodies against TSHR and IGF-1R on orbital fibroblasts were discovered to be a significant cause of TED [7, 9]. The activation of the TSHR and IGF-1R complex is a very important aspect of TED, which leads to an abnormal immunoproliferative response in the orbit, causing hypertrophy of the extraocular muscles and an increase in intraorbital adipose tissue, resulting in various clinical manifestations such as proptosis, diplopia, pain, and compressive optic neuropathy [7, 10].

Teprotumumab (trade name TEPEZZA™) is a fully human IgG1 monoclonal antibody that specifically targets the IGF-1R. It received FDA approval for marketing on January 21, 2020, making it the first and only drug approved by the FDA for the treatment of TED. The pharmacokinetic parameters of teprotumumab were found to be linear in patients with TED. The drug exhibited a clearance of 0.334 L/day, distribution into central and peripheral compartments with volumes of 3.9 L and 4.2 L, respectively, and a half-life of 19.9 days. Furthermore, the steady-state area under the concentration-time curve, peak concentration, and trough concentration were measured to be 131 mg·h/mL, 643 *μ*g/mL, and 157 *μ*g/mL, respectively [11].

Teprotumumab outperforms intravenous methylprednisolone (IVMP) in improving both proptosis and diplopia [12]. As per the recommendations of the American Thyroid Association and the European Thyroid Association, teprotumumab is considered a preferred therapy for patients with active moderate-to-severe TED who exhibit significant proptosis and/or diplopia [13]. But for glucocorticoid- resistant patients, teprotumumab has not been evaluated in this setting. In addition, another consensus reached by Douglas et al. [2] suggests that teprotumumab is suitable for patients with a CAS score of <3, lid retraction of ≥2, and mild or early optic neuropathy, with close clinical observation. As time goes on, some studies have suggested that teprotumumab significantly reduces ocular proptosis and diplopia and improves quality of life; the current recommendation is for a second-line option by the European Group on Graves' Orbitopathy [2]. However, some studies have linked teprotumumab to side effects such as inflammatory bowel disease, hyperglycemia, and hearing loss [14]. In this paper, we conducted a comprehensive systematic review and meta-analysis of all available randomized controlled trials (RCTs) of teprotumumab in individuals with TED to assess the clinical response and adverse events associated with its use.

## 2. Methods

### 2.1. Study Search and Selection

All clinical studies were identified through a systematic review of the literature in PubMed, Embase, and the Cochrane Library from inception to May 25, 2022, and using the search terms: “teprotumumab” [MeSH] OR “HZN-001” OR “RV 001” OR “TEPEZZA™.” Only RCTs comparing the clinical efficacy and adverse effects of teprotumumab and placebo in the treatment of adult patients with TED and studies in English were included. Following the completion of the search, EndNote X8 was used to remove duplicate records. The title and abstract of the remaining records were independently read by two researchers for preliminary screening. The full text of publications that potentially met the inclusion criteria was also reviewed. Any disagreements arising during the reading process were resolved through discussion involving a third researcher. The relevant information, including the study's basic characteristics, baseline patient characteristics, intervention measures, and efficacy and safety outcomes, was extracted from all the included studies. Two investigators independently collected the data using a standardized form. If any discrepancies arose during the data extraction process, consensus was reached through discussion with a third investigator. Finally, the Cochrane Collaboration bias assessment tool was employed to evaluate the risk of bias in the included studies [15].

### 2.2. Outcome Indicators

Proptosis response, proptosis, diplopia response, overall response, Graves' ophthalmopathy-specific quality of life (GO-QOL), and clinical activity score (CAS) were the primary outcome indicators. The proptosis response was defined as a reduction in proptosis of ≥2 mm from baseline; the overall response was defined as a reduction of ≥2 points in the CAS and a reduction in proptosis of ≥2 mm; a CAS of 0 or 1 was defined as indicating no or minimal inflammation; and a diplopia response was defined as a reduction in diplopia of ≥1 Bahn–Gorman grade from baseline [16–18]. The secondary outcome indicators included the incidence of any adverse events (AEs), several adverse events (SAEs), and other AEs.

### 2.3. Statistical Analysis

The statistical analyses were performed using Review Manager 5.3 software. Mean differences (MDs) were calculated for continuous measurement data, while odds ratios (ORs) were calculated for dichotomous variables, both with 95% confidence intervals (CIs). To assess between-study heterogeneity, the chi-square test and the *I*^2^ test were utilized for quantification. A fixed-effect model was applied when *I*^2^ was less than 50% and the *P* value was greater than 0.1 [19]. Conversely, a random-effect model was used when *I*^2^ was greater than 50% and the *P* value was greater than 0.1. Furthermore, we conducted sensitivity analyses and subanalyses to explore the potential sources of heterogeneity. A significance level of *P* < 0.05 was considered statistically significant.

## 3. Results

### 3.1. Study Identification and Study Characteristics

**Figure 1:**
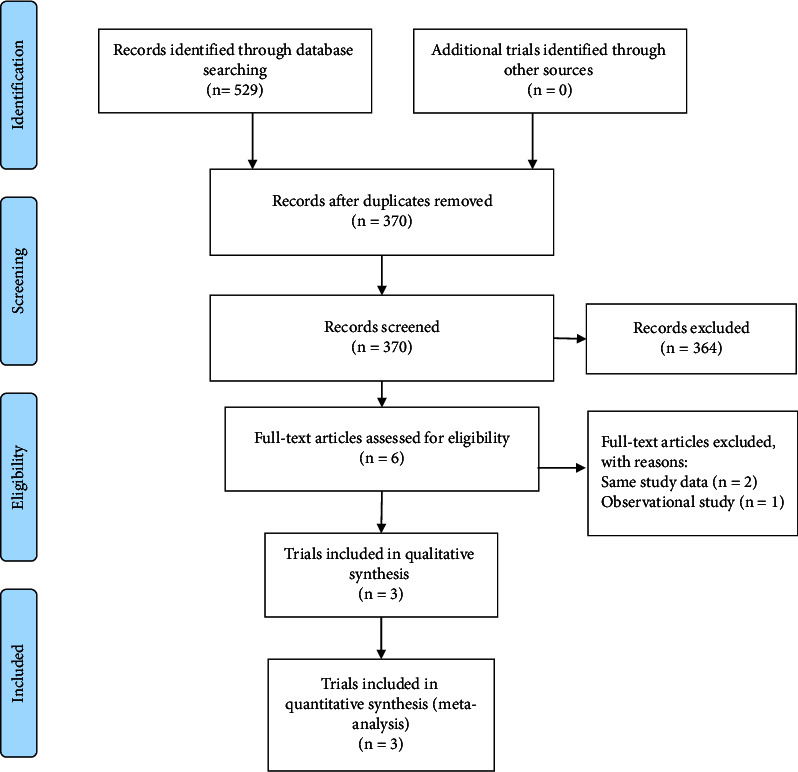
Flowchart of the study selection process.

**Figure 2:**
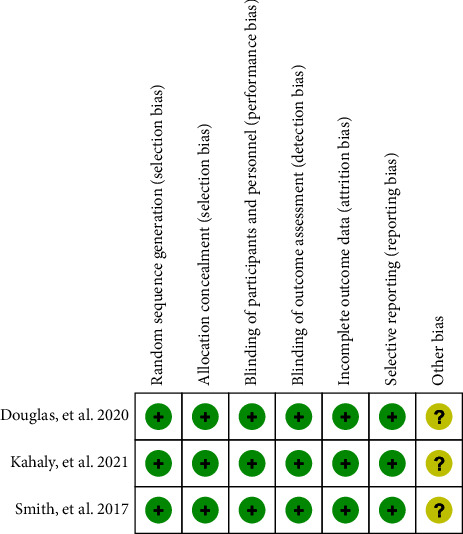
Risk of bias per study and domain.

**Figure 3:**
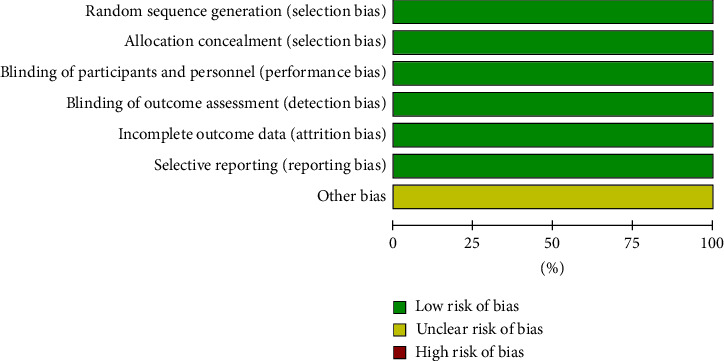
Risk of bias graph.

Initially, a total of 529 articles were identified through searches in the Cochrane Library (*n* = 29), PubMed (*n* = 180), and Embase (*n* = 320). After removing duplicate literature (*n* = 159), the remaining articles were subjected to further screening. Ultimately, only three studies that met the inclusion criteria were included [16–18]. The included studies involved a total of 341 patients with thyroid eye disease. All of the selected studies were published in English. The process of literature screening is illustrated in Figure 1. Detailed characteristics of the included studies and baseline demographic information can be found in Table 1. The results of the quality assessment are presented in Figures 2 and 3.

Overall, the teprotumumab treatment group and placebo group consisted of 168 and 173 patients, respectively. Of these, females accounted for 68.5% and 77.5% of the population in the teprotumumab and placebo groups, respectively. In the teprotumumab and placebo groups, patients were 51.6 ± 11.5 and 51.5 ± 13.1 years old, respectively. All patients took part in the three clinical trials, all of which had a CAS of at least 4, and prior treatment with at least 1 g of methylprednisolone was permitted with a minimum washout of 4 weeks [18] or 6 weeks [16, 17]. All the patients received teprotumumab intravenous infusions, one every 3 weeks, starting with an initial dose of 10 mg/kg of body weight, followed by 20 mg/kg, for a total of eight infusions [16–18].

### 3.2. Clinical Efficacy

**Figure 4:**
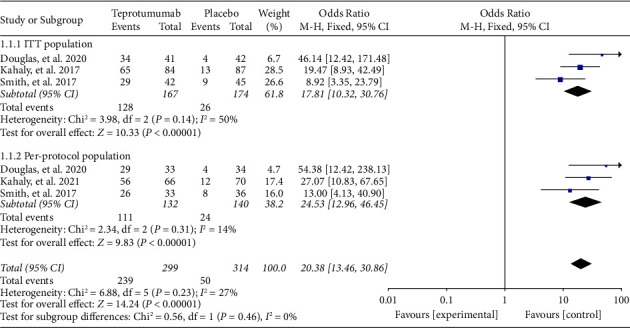
Meta-analysis results from clinical trials for proptosis response.

In the outcome measure in the ITT population, a proptosis response was observed in 71.86% (128/167) of patients who received teprotumumab and in 14.94% (26/174) of patients who received a placebo. Similarly, in the per-protocol population, a proptosis response was observed in 84.09% (111/132) of patients who received teprotumumab and in 17.14% (24/140) of patients who received a placebo. Overall, the integrated proptosis response was significantly higher in the teprotumumab group compared to the placebo group in both the ITT population (OR = 17.81, 95% CI = [10.32, 30.76], *I*^2^ = 50%, *P* < 0.00001) and per-protocol population (OR = 24.53, 95% CI = [12.96, 46.45], *I*^2^ = 14%, *P* < 0.00001) as depicted in Figure 4.

**Figure 5:**
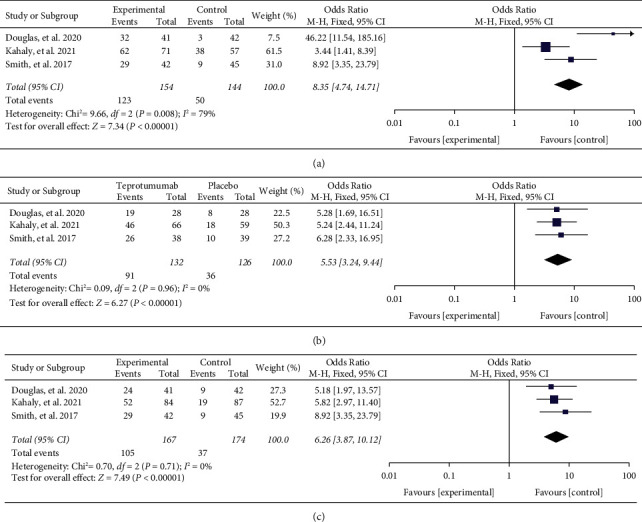
Meta-analysis results from clinical trials for overall response (a), diplopia response (b), and CAS of 0 or 1 (c).

Patients treated with teprotumumab showed significant improvement in overall response and diplopia response compared to the placebo group. In the pooled analysis of three studies, 73.65% of 167 patients in the teprotumumab group demonstrated improvement in overall response, as measured by CAS plus proptosis improvement, compared to 13.79% of 174 patients in the placebo group (OR = 8.35, 95% CI = [4.74, 14.71], *I*^2^ = 79%, *P* < 0.00001). Similarly, the diplopia response rate was higher in the teprotumumab group, with 68.94% (91/132) of patients improving by one grade or more, compared to 28.57% (36/126) of patients in the placebo group (OR = 5.53, 95% CI = [3.24, 9.44], *I*^2^ = 0%, *P* < 0.00001) as illustrated in Figure 5.

The CAS is an important criterion used to assess disease activity in thyroid-associated ophthalmopathy on a 7-point scale. A CAS score of ≥3 indicates active disease. In all three clinical trials, all participants had a baseline CAS score of at least 4. Following treatment with teprotumumab, the CAS showed a significant reduction; the proportion of sufferers with a CAS of 0 or 1 was 62.87% in the teprotumumab group and 21.26% in the placebo group. CAS of 0 or 1 had a significant difference from placebo (OR = 6.26, 95% CI = [3.87, 10.12], *I*^2^ = 0%, *P* < 0.00001).

**Figure 6:**
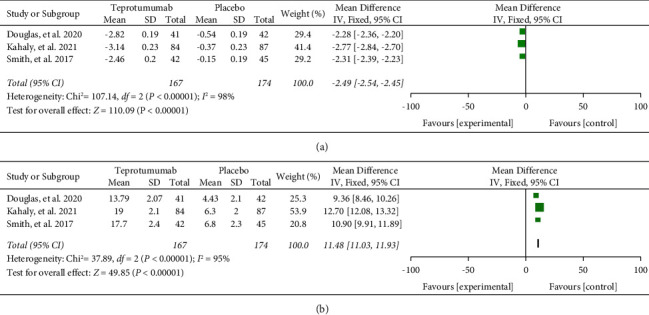
Meta-analysis results from clinical trials for change from baseline in proptosis (a) and GO-QOL (b).

Moreover, patients treated with teprotumumab had a greater improvement in proptosis (MD = −2.49, 95% CI = [−2.54, −2.45], *I*^2^ = 98%, *P* < 0.00001) and GO-QOL (MD = 11.48, 95% CI = [11.03, 11.93], *I*^2^ = 95%, *P* < 0.00001) as demonstrated in Figure 6. In the sensitivity analysis, according to the data from the study conducted by Kahaly et al. [17], the heterogeneity of proptosis and GO-QOL change from baseline decreased from 98% to 0% and 95% to 80%, respectively.

### 3.3. Safety

A significant difference was found between teprotumumab and placebo for the risk of AEs (OR = 0.93, 95% CI = 0.80–1.08, *I*^2^ = 75%) and SAEs (OR = 5.12, 95% CI = [1.44, 18.13], *I*^2^ = 0%, *P* = 0.01) (Table 2). The risk estimates and associated statistics for the outcomes of alopecia, diarrhea, fatigue, and headache are as follows: alopecia (OR = 1.71, 95% CI = [0.84, 3.49], *I*^2^ = 0%, *P* = 0.14); diarrhea (OR = 1.52, 95% CI = [0.74, 3.12], *I*^2^ = 0%, *P* = 0.25); fatigue (OR = 2.36, 95% CI = [0.93, 6.00], *I*^2^ = 0%, *P* = 0.07); and headache (OR = 1.21, 95% CI = [0.54, 2.71], *I*^2^ = 0%, *P* = 0.64). In the pooled analysis of three RCTs, all-cause mortality did not occur during the trial. There have been more AEs and SAEs in the three clinical trials, including hyperglycemia, hearing impairment, rash, onychoclasis, stomatitis, amenorrhea, dizziness, cough, upper abdominal pain, influenza, pneumothorax, visual-field defect, paresthesia, weight loss, optic neuropathy, Hashimoto's encephalopathy, inflammatory bowel disease, and urinary retention. We did not conduct meta-analyses for these AEs because the events were rare, such as hyperglycemia, and a total of 22 participants across all trials experienced severe hyperglycemia, defined as an event requiring assistance: 13 with teprotumumab (*n* = 168); 9 with a placebo (*n* = 173).

## 4. Discussion

TED is an organ-specific autoimmune disease that manifests as infiltrative lesions in the posterior and periorbital ocular tissues. It represents one of the most frequent extra-thyroidal manifestations of thyroid disease, and it significantly affects the quality of life of affected individuals [5]. Corticosteroids are considered the first choice of treatment for this disease, but their use is limited by the long-term toxicity of large amounts of corticosteroids, including peptic ulcers and osteoporosis [1, 3, 9]. In addition, some immunosuppressive drugs are used to treat the disease, but their use is limited by their toxicity or efficacy [1]. Teprotumumab is an IGF-1R monoclonal antibody available for TED therapy and can inhibit the interaction with TSHR signaling by blocking the activation of IGF-1R [7, 20]. The results of the present study demonstrate that the use of teprotumumab yielded a greater integrated proptosis response compared to placebo, and the overall response, diplopia response, CAS, proptosis, and GO-QOL were significantly improved in patients with teprotumumab vs. placebo.

Overall, in the ITT population and per-protocol population, the integrated proptosis response to teprotumumab was greater than placebo, respectively. Teprotumumab (OR = 8.92, 95% CI = [2.51, 31.77]) produced significantly better improvement than no treatment, according to Zhou et al. [21]. 71.86% (128/167) of patients who received teprotumumab and 14.94% (26/174) of patients who received a placebo had a proptosis response in the ITT population. The proptosis response was maintained in 29 of 32 patients (90.6%) in the OPTIC-X Study, according to authors [22]. As defined by the CAS plus prognosis improvement, 73.65% of patients in the teprotumumab group vs. 13.79% of patients in the placebo group improved their overall response in our study. Similarly, the diplopia response rate (improved by one grade or more) was higher with teprotumumab vs. placebo (68.94% vs. 28.57%).

In this study, we observed improved soft tissue inflammation, as measured by CAS, in all participants across three clinical trials. Specifically, participants with a CAS of ≥4 points on a 7-point scale in the more severely affected eye (study eye) demonstrated the improvement. After receiving teprotumumab, the CAS showed a significant reduction; the proportion of sufferers with a CAS of 0 or 1 was 62.87% in the teprotumumab group and 21.26% in the placebo group. Some studies found teprotumumab significantly reduced inflammatory signs, and CAS reduction was significantly greater for teprotumumab than placebo at week 24 (phase 2: −3.43 vs. −1.85 points; phase 3: −3.7 vs. −2.0 points); it had a CAS of 0 or 1 compared with placebo (phase 2: 69% vs. 21%; phase 3: 59% vs. 21%) [14]. In addition, a CAS reduction of at least 3 points was more frequent with teprotumumab vs. placebo [14]. In the placebo arm, although the mean CAS in the study eye decreased over time, proptosis and diplopia did not show any changes in either orbit [23]. On the other hand, patients treated with teprotumumab exhibited significant reductions in proptosis, CAS, and diplopia in both orbits [23]. This may be related to the fact that teprotumumab can reduce the soft tissue volume in the orbit [24].

Similar to the clinical outcome findings, patients who received teprotumumab showed a significant improvement in proptosis, with a decrease of −2.49 mm (95% CI = [−2.54, −2.45]). The difference in proptosis treatment between IVMP and teprotumumab favored teprotumumab, with a reduction of −2.31 mm (95% CI = [−3.45, −1.17]). Treatment with teprotumumab was found to be more favorable than IVMP, with an OR of 2.32 (95% CI = [1.07, 5.03]). However, it is important to note that these findings are based on a nonrandomized comparison, and further randomized trials are necessary to establish clinical superiority between the two treatments [12]. The evaluation of quality of life after treatment in patients with TED was performed using the GO-QOL questionnaire. A clinically significant change in GO-QOL is defined as a difference of ≥6 points in one or both subscales. In our study, patients receiving teprotumumab demonstrated a greater improvement in GO-QOL, with a difference of 11.48 points (95% CI = [11.03, 11.93]).

As for safety, a significant difference was found between teprotumumab and placebo for the risk of AEs and SAEs. The incidence of AEs and SAEs was 79.76% and 8.3%, respectively. In contrast, the OPTIC-X Study by Douglas et al. [22] discovered that all AEs were mild or moderate, with no patients experiencing a severe adverse event. In addition, the risk of alopecia, diarrhea, fatigue, and headache was similar between teprotumumab and placebo. The risk of muscle spasm, nausea, dry skin, and dysgeusia was, however, slightly higher with teprotumumab than with placebo. Teprotumumab's ability to reduce muscle volume may explain the increased risk of muscle spasm [24]. Also, all-cause mortality did not occur during the trial.

To the best of our knowledge, this is the first systematic review and meta-analysis that comprehensively evaluates the available evidence regarding the use of teprotumumab. While we included RCTs, it is important to acknowledge several limitations of the current study. First, the RCTs included in our analysis were sponsored by the pharmaceutical industry, which may introduce potential bias and should be interpreted with caution. Second, the number of included studies is limited, and RCTs have generally been conducted with small and selective populations of patients with TED, leading to a potential risk of selection bias. Finally, the comparison of teprotumumab with other therapy drugs, such as tocilizumab, rituximab IV steroids, or methylprednisolone, that are recommended by the American Thyroid Association and the European Thyroid Association was not included in our analysis in terms of their efficacy on proptosis or diplopia. Therefore, it is necessary to acquire more data and analyze these findings in future trials to further evaluate the efficacy and safety of teprotumumab and make more robust conclusions.

In summary, patients treated with teprotumumab demonstrated greater improvement in proptosis response, proptosis, diplopia response, overall response, GO-QOL, and CAS. However, it is important to acknowledge that comparisons are challenging due to the limited number of patients receiving teprotumumab and the absence of comparison with other effective therapeutic options. Future studies with long-term observation are necessary to assess the durability of the response. In addition, the risk of gastrointestinal adverse reactions and muscle spasm could warrant caution when receiving teprotumumab treatment.

## Figures and Tables

**Table 1:** Study characteristics and baseline demographic characteristics in meta-analysis.

Study, year published	Intervention	No. of patients	Study duration	Therapy duration	Study population	Study design	Study site	Female (%)	Mean ± S.D. age (years)
Smith et al. [16], 2017	Teprotumumab	43	July 2013 and September 2015	24 weeks	18–75 years	Phase 2	15 sites in the United States and Europe	28 (65)	51.6 ± 10.6
	Placebo	44						36 (82)	54.2 ± 13.0

Douglas et al. [18], 2020	Teprotumumab	41	October 2017 and August 2018	24 weeks	18–80 years	Phase 3	13 sites in the United States and Europe	29 (71)	51.6 ± 12.6
	Placebo	42						31 (74)	48.9 ± 13.0

Kahaly et al. [17], 2021	Teprotumumab	84	October 2017 and August 2018	24 weeks	18–75 years	Phase 3	28 sites in Europe and the USA	58 (69)	51·5 ± 11·6
	Placebo	87						67 (77)	51·4 ± 13·1

**Table 2:** The results of safety in meta-analysis.

Outcomes	Participants		*I * ^2^ (%)	Effect estimate	*P* value
	Dorzagliatin arm	Comparator arm			
Any AEs	134/168	121/172	0	1.66 [1.01, 2.73]	0.05
SAEs	14/168	3/172	0	5.12 [1.44, 18.13]	0.01
Muscle spasm	42/168	12/172	0	4.48 [2.26, 8.89]	<0.0001
Nausea	28/168	16/172	0	1.95 [1.01, 3.75]	0.05
Alopecia	22/168	14/172	0	1.71 [0.84, 3.49]	0.14
Diarrhea	20/168	14/172	0	1.52 [0.74, 3.12]	0.25
Fatigue	15/168	7/172	0	2.36 [0.93, 6.00]	0.07
Headache	14/168	12/172	0	1.21 [0.54, 2.71]	0.64
Dry skin	14/168	0/172	0	11.54 [2.13, 62.57]	0.005
Dysgeusia	14/168	0/172	0	11.54 [2.13, 62.57]	0.005

## Data Availability

All the data supporting this systematic review are from previously reported studies and datasets, which have been cited. The processed data are available from the corresponding author upon request.
